# Determinants of urgent start dialysis in a chronic kidney disease cohort followed by nephrologists

**DOI:** 10.1186/s12882-023-03222-1

**Published:** 2023-06-27

**Authors:** Amin Tachikart, Clément Vachey, Charline Vauchy, Caroline Savet, Didier Ducloux, Cécile Courivaud

**Affiliations:** 1 Department of Nephrology, Dialysis and Renal Transplantation, Univ Hospital of Besançon, Besançon, France; 2 Agence de la Biomédecine, REIN Registry, Saint Denis La Plaine Cedex, Paris, France

**Keywords:** Advanced chronic kidney disease, Dialysis access, Urgent dialysis start, Survival, France

## Abstract

**Background:**

The French Renal Epidemiology and Information Network (REIN) registry collect dialysis initiation context for each patient starting dialysis with a flawed definition of urgent start dialysis (USD). The main objective of this study was to identify factors associated with USD in patients regularly followed-up by a nephrologist using a classification of USD considering the preparation to renal replacement therapy.

**Methods:**

This retrospective cohort study included adult patients who started dialysis between 2012 and 2018 in the Franche-Comté region of France after a minimum of two nephrology consultations. We classified dialysis initiation context as follows: USD for patients with no dialysis access (DA) created or planned, unplanned non urgent start dialysis (UNUSD) for patients starting with a recent or non-functional DA and planned start dialysis (PSD) for those starting with a functional and mature DA.

**Results:**

Four hundred and sixty-five patients met inclusion criteria. According to REIN registry, 94 (20.3%) patients were urgent starters (US) whereas with our classification 80 (17.2%) and 73 (15.7%) where respectively US and unplanned non urgent starters (UNUS). The factors independently associated with USD in our classification were: stroke (odds ratio(OR) = 2.76, 95% confidence interval (95%CI)=[1.41–5.43]), cardiac failure (OR = 1.78, 95%CI=[1.07–2.96]) and the number of nephrology consultations prior dialysis onset (OR = 0.73, 95%CI=[0.64–0.83]). Thirty-one patients died during the first year after dialysis start. According to our classification, we observed significantly different survival probabilities: 95.7%, 89.5% and 83.4% respectively for planned starters, UNUS and US (p = 0.001).

**Conclusion:**

The two factors independently associated with USD were cardiac failure and stroke.

**Supplementary Information:**

The online version contains supplementary material available at 10.1186/s12882-023-03222-1.

## Introduction

Kidney Failure (KF) prevalence is increasing steadily. In France, according to data from the French Renal Epidemiology and Information Network (REIN) registry, 11 343 patients started chronic renal replacement therapy (RRT) in 2018 (95.9% on dialysis and 4.1% with preemptive kidney transplantation (KT)). Over the past decades, survival among dialysis patients appears to improve. However a substantial and stable proportion of KF patients start RRT in an emergency context which is a major risk factor of mortality during the first year of dialysis [1]. Indeed, in 2018 in France, for about 30% of the patients, dialysis was started in an emergency context (29.9% on hemodialysis (HD) and 6% on peritoneal dialysis (PD)). Among them, 87% started RRT with a central venous catheter (CVC).

Dialysis start in emergency in REIN registry is defined as follows: “the first dialysis session (hemodialysis or peritoneal dialysis) is performed immediately (< 24 h) after an evaluation by a nephrologist due to a vital risk, notably following threatening hyper hydration, hyperkalemia, acidosis, poorly tolerated anemia, uremic pericarditis or uremic confusion. The presence of only one of these criteria defines urgency. This notion does not exclude acute decompensation despite early preparation by a nephrologist”.

This definition has some weakness. First, this empirical 24-hour limit may not include some patients who might have a similar clinical profile to those starting in emergency conditions. Moreover, it does not take into account the preparation to RRT that the patients may have benefit, especially the creation of a permanent dialysis access (DA). Indeed, in 2018, 13% of urgent starters (US) had a functional DA. A few studies showed that a native arteriovenous fistula (NAVF) was associated with a better survival that the use of CVC or a prosthetic fistula (PAVF) [2]. Finally, this definition probably gathers together patients with very different profiles and different outcomes, which may have led to bias in the crude interpretation of the results.

A few studies have highlighted the fact that late referral to a nephrologist is associated with an increased risk of urgent start dialysis (USD) and increased mortality [3, 4]. In 2018 REIN report, among US, 38.3% reported no consultation during the year preceding dialysis initiation whereas 42% had at least 3 consultations. In patients regularly followed-up by a nephrologist, factors associated with an USD are currently unclear. While nephrologists aim for optimal conditions for dialysis start, in terms of nutrition, hemoglobin level, phosphocalcic metabolism, fluid overload, DA… [5]; it was shown that USD made lose all the benefit of early referral [6]. Unfortunately, for now, few data allow us to identify patients at risk of USD and better anticipate dialysis onset in this population.

The main objective of this study was to identify factors associated with USD in patients regularly followed-up by a nephrologist using a classification of USD considering the preparation to RRT. The secondary objectives were to compare characteristics and outcomes of US according to the REIN registry classification and ours, to identify factors associated with mortality during the first year of dialysis.

## Patients and methods

### 1/ Study population

This work is a retrospective, epidemiological, analytical cohort study in the Franche-Comté region of France. We included patients who started dialysis in one of the four main centers of the region (Besançon, Belfort-Montbéliard, Vesoul and Dole) from January 1st, 2012 to December 31st, 2018. To be included, patients had to be of legal age (more than eighteen years old) and have had at least two nephrology consultations during the year preceding dialysis. Exclusion criteria included: a return to dialysis after a KT, patients who started dialysis because of heart failure refractory to drug therapy.

### 2/ Data collection

First, few data were extracted from REIN registry, the first RRT modality: PD *versus* (vs.) HD, with its date of starting and its condition (emergency vs. no emergency). We also collected the date of death, KT, dialysis withdrawal whatever the cause or technique switch. We used data from 2012, January 1st to 2018, December 31st .

Secondarily we completed with data extracted from each patient’s medical file including patient’s medical history: heart failure (HF), ischemic cardiomyopathy, body mass index (BMI), stroke with or without sequelae, neoplasia with or without remission, smoking cessation or active smoking, peripheral arteriopathy, chronic obstructive pulmonary disease (COPD), immunosuppression (IS) (including chemotherapy and corticotherapy) the number of nephrology consultations with corresponding date, creatinine and estimated glomerular filtration rate (eGFR) on the day of the consultation.

Regarding the DA, we collected the type of the first DA (NAVF, PAVF, CVC or PD catheter) and its date of creation.

At dialysis onset we collected: the number of antihypertensive drugs, the use of a renin-angiotensin system blocker (RASB), the use of an erythropoietin stimulating agent (ESA) and the presence of a fluid overload. The following biological parameters: urea, creatinine and corresponding eGFR (calculated with MDRD formula), sodium, potassium, bicarbonate, calcium, phosphorus, albumin, hemoglobin, C-reactive protein (CRP) were collected as well.

### 3/ Emergency context assessment

In addition to the REIN registry definition of USD, we developed another classification with 3 groups of patients defined as follows:

- USD: defined as the absence of prior creation of a DA and no creation planned within a period of a month when the patient starts dialysis.
- Unplanned non urgent start dialysis (UNUSD) corresponds to the following situations:
- Patients who start with a functional DA but recently created that is to say: less than 4 weeks before dialysis start for an NAVF, 2 weeks for PAVF and 1 week for a PD catheter.
- Patients who had the creation of DA but which is not functional at dialysis start because of complication and consequently require a CVC.
- Patients who start with a CVC while waiting for a KT (with deceased or living donor) and have no project of other DA because the period of dialysis is expected to be short.
- The planned start dialysis (PSD) corresponds to the initiation of dialysis in optimal conditions for the patient with a DA of more than four weeks for NAVF, two weeks for PAVF, and more than one week for PD catheters and CVC for those with no DA possible.

### 4/ Statistical analysis

Numeric variables were described as mean and standard deviation (SD) or median and interquartile range (IQR); categorical variables were described in terms of numbers and percentages. Missing data are reported as appropriate. Univariable analyses were performed using Student t-test or Wilcoxon-Mann-Whitney test for continuous variable. Categorical data were analyzed using the Chi-square or Fisher’s exact test. Comparisons between the 3 groups were performed using the Kruskal-Wallis test. Multivariable analyses were performed with a logistic regression.

The Kaplan Meier method was used to estimate patient survival during the first year of dialysis and then a comparison between each group was performed using the log-rank test. Multivariable survival analysis was performed with semi-parametric Cox proportional-hazards model. Patient follow-up was censored after one year of dialysis. Patients who benefit from a KT or a dialysis withdrawal during the first year were censored at this date. All tests were two-sided and P values < 0.05 were considered statistically significant. Statistical analyses were performed using SAS version 9.4 (SAS Institute Inc, Cary, North Carolina, USA).

## Results

### 1/ Population characteristics

In the Franche-Comté region of France, between January 1st, 2012 and December 31st, 2018, 1 066 patients started chronic dialysis, including 981 adults who started in one of the four participating centers. Of these 348 (35.5%) had either no previous nephrology consultation or an unknown number of consultations (that could not be found because of a change in medical record format in one center during the study period), 57 (5.8%) had only 1 consultation. Among the 576 remaining patients, 111 were not included on account of missing biology data. Finally, 465 were included for analysis. Among these 465 patients, 309 (70.2%) were men and the median age was 70.0 (IQR: 59–79). The initial dialysis technique was PD for 138 (29.7%) patients and the median eGFR at dialysis start was 8.5 mL/min/1.73 m² (IQR: 6.7–10.7).

According REIN registry, USD concerned 94 patients (20.3%) and this information was missing for 3 patients (Table 1). The clinical factors associated with USD according to REIN definition were: diabetes (53.2% in US vs. 39.1%, p = 0.01), cardiac failure (58.5% vs. 39.1%, p < 0.0003) stroke (20.2% vs. 8.4%, p = 0.001). The number of visits was significantly higher in the non-urgent group (4.5 ± 1.9 for US vs. 5.7 ± 2.0, p < 0.0001). Moreover, non-urgent starters were more likely to have RASB therapy at dialysis initiation (50.3% and 37.6% for planned starters (PS) and US respectively, p = 0.03). Fewer US started RRT on PD (1.1% vs. 37.2%, p < 0.0001) and they were more likely to start dialysis with a CVC (83.0% vs. 21.0%, p < 0.0001).

According our classification, 17.2% patients were considered as US, 15.7% had a UNUSD and 67.1% had a PSD (Table 1). Concerning factors associated with USD, we observed similar results than those previously described. Cardiac failure was associated with USD affecting 49.4% of US, 41.1% of unplanned non urgent starters (UNUS) and 32.4% of PS (p = 0.04). Stroke was also associated with USD concerning 21.0% of US, 11.0% of UNUS and 8.4% of PS (p = 0.004). Nevertheless, contrary to REIN classification, diabetes repartition was less clear since 44.4% of US were diabetic, 38.6% of PS and more than 53.4% of UNUS (p = 0.05). Cancer, COPD, smoking status, peripheral arteriopathy, ESA use and IS were not associated with USD whatever the classification.

Comparing the 2 classifications (Table 2), 16% of the patients classified in REIN as US were considered, with our definition, as having a PSD. Conversely, 5.7% of the patients classified as non-urgent starters in REIN were classified as US in our classification.

**Table 1:** Characteristics of the population according to REIN classification (n = 462*) and our classification (n = 465): (n(%) or mean ± SD)

	USD according REIN classification			Dialysis start according our classification			
	Yes n = 94 (20.3)	No n = 368 (79.5)	p value	Urgent n = 80 (17.2)	Suboptimal n = 73 (15.7)	Planned n = 312 (67.1)	p value
Sex (male)	66 (70.2)	241 (65.5)	0.39	52 (65.0)	48 (65.8)	210 (67.3)	0.91
Age (years)	67.0 ± 14.4	67.2 ± 14.7	0.91	66.3 ± 15.1	68.6 ± 12.4	67.0 ± 14.9	0.82
BMI (kg/m^2^, 62**)	26.2 ± 6.8	26.7 ± 7.2	0.43	25.9 ± 8.1	27.6 ± 8.4	26.5 ± 6.5	0.31
Initial nephropathy							
- Chronic glomerulonephritis	15 (15.8)	44 (12.0)		14 (17.5)	3 (4.1)	42 (13.5)	
- Diabetic nephropathy	27 (28.4)	96 (26.2)		20 (25.0)	25 (34.2)	78 (25.1)	
- Interstitial chronic nephropathy	1 (1.1)	12 (3.3)		2 (2.5)	3 (4.1)	9 (2.9)	
- Nephroangiosclerosis	29 (30.5)	96 (26.2)		20 (25.0)	21 (28.8)	84 (27)	
- Polycystic kidney disease	5 (5.3)	40 (10.9)		6 (8.0)	6 (8.2)	34 (10.9)	
- Others and undetermined nephropathy	8 (1.7)	46 (10.0)		10 (2.2)	8 (1.7)	36 (7.7)	
- Urological nephropathy	9 (9.5)	34 (9.3)		9 (11.1)	6 (8.2)	29 (9.3)	
History							
- Ischemic cardiomyopathy	8 (8.5)	39 (10.6)	0.55	36 (45.0)	39 (53.4)	120 (38.4)	0.05
- Diabetes	55 (58.5)	144 (39.1)	0.01	7 (8.8)	8 (11.0)	32 (10.3)	0.89
- Cardiac failure	50 (53.2)	121 (32.9)	0.0003	40 (50.0)	30 (41.1)	101 (32.4)	0.01
- Stroke	19 (20.2)	31 (8.4)	0.001	17 (21.3)	8 (11.0)	26 (8.3)	0.004
- Cancer	20 (21.3)	82 (22.3)	0.83	19 (23.8)	14 (19.2)	71 (22.8)	0.76
- Current smoker	10 (10.6)	45 (12.2)	0.67	9 (11.3)	6 (8.2)	41 (13.1)	0.51
- Peripheral arteriopathy	28 (29.8)	79 (21.5)	0.09	17 (21.3)	20 (27.4)	71 (22.8)	0.63
- IS	18 (19.2)	54 (14.7)	0.29	18 (22.5)	12 (16.4)	42 (13.5)	0.13
- COPD	18 (19.2)	46 (12.5)	0.10	14 (17.5)	10 (13.7)	40 (12.8)	0.56
Number of nephrology consultations prior dialysis start	4.5 ± 1.9	5.7 ± 2.0	< 0.0001	4.2 ± 1.8	4.7 ± 2.0	5.9 ± 2.4	< 0.0001
Dialysis start with a CVC	78 (83.0)	65 (21.0)	< 0.0001	80 (100)	59 (83.1)	6 (2.3)	< 0.0001
Peritoneal dialysis *vs.* HD	1 (1.1)	137 (37.2)	< 0.0001	0	5 (6.9)	133 (42.6)	< 0.0001
ESA (2**)	74 (79.6)	295 (80.4)	0.86	37 (45.6)	23 (32.9)	158 (51.3)	0.02
RASB (7**)	35 (37.6)	182 (50.3)	0.03	65 (80.2)	57 (78.1)	249 (80.0)	0.86
Number of antihypertensive drugs (7**)	2.6 ± 1.4	2.7 ± 1.3	0.79	2.7 ± 1.5	2.5 ± 1.4	2.7 ± 1.3	0.61

*Starting conditions were missing for 3 patients, ** missing data; BMI: body mass index, COPD: chronic obstructive pulmonary disease, CVC: central venous catheter, IS: immunosuppression, HD: hemodialysis, ESA: erythropoietin stimulating agent, RASB: renin-angiotensin-system blocker USD: urgent start dialysis

### 2/ Biological parameters at dialysis initiation

According our classification, the biological analysis of patients starting dialysis objectively showed significant differences on all biological parameters with better parameters in PS, except for CRP and serum calcium levels (Table 1 in supplementary data). Indeed, PS had a better hemoglobin level (p < 0.0001), a lower serum phosphate level (p < 0.0001), a higher albumin level (p < 0.0001) and a higher serum bicarbonate level (p = 0.0004). Concerning eGFR, we observed significantly lower eGFR in US (7.8 mL/min/1.73 m² ±3.1 for US vs. 8.5 ± 2.6 and 9.3 ± 3.2 for UNUS et PS respectively, p = 0.0002). In addition, patients starting in emergency had more frequently overload fluid (72.5% of US, 54.6% of suboptimal starters and 43.8% of PS, p = 0.0001).

Similar results were observed with REIN classification (data not shown) with especially a mean eGFR of 7.6 mL/min/1.73 m² ±3.0 for US vs. 9.3 ± 3.1 for PS (p < 0.0001).

### 3/ Risk factors of USD

In multivariable analyses (Table 2 in supplementary data), two factors were independently associated with USD: cardiac failure (OR = 1.78, 95% confidence interval (CI) [1.07–2.96], p = 0.003) and stroke (OR = 2.76, 95%CI [1.41–5.43], p = 0.02). The number of consultations during the year prior dialysis start, considered as a continuous variable, was associated with a significant reduction of USD risk (OR = 0.73; 95%CI [0.64–0.83], p = 0.02). There was not any other factor independently associated with USD.

### 4/ Mortality during the first year of dialysis

**Fig. 1:**
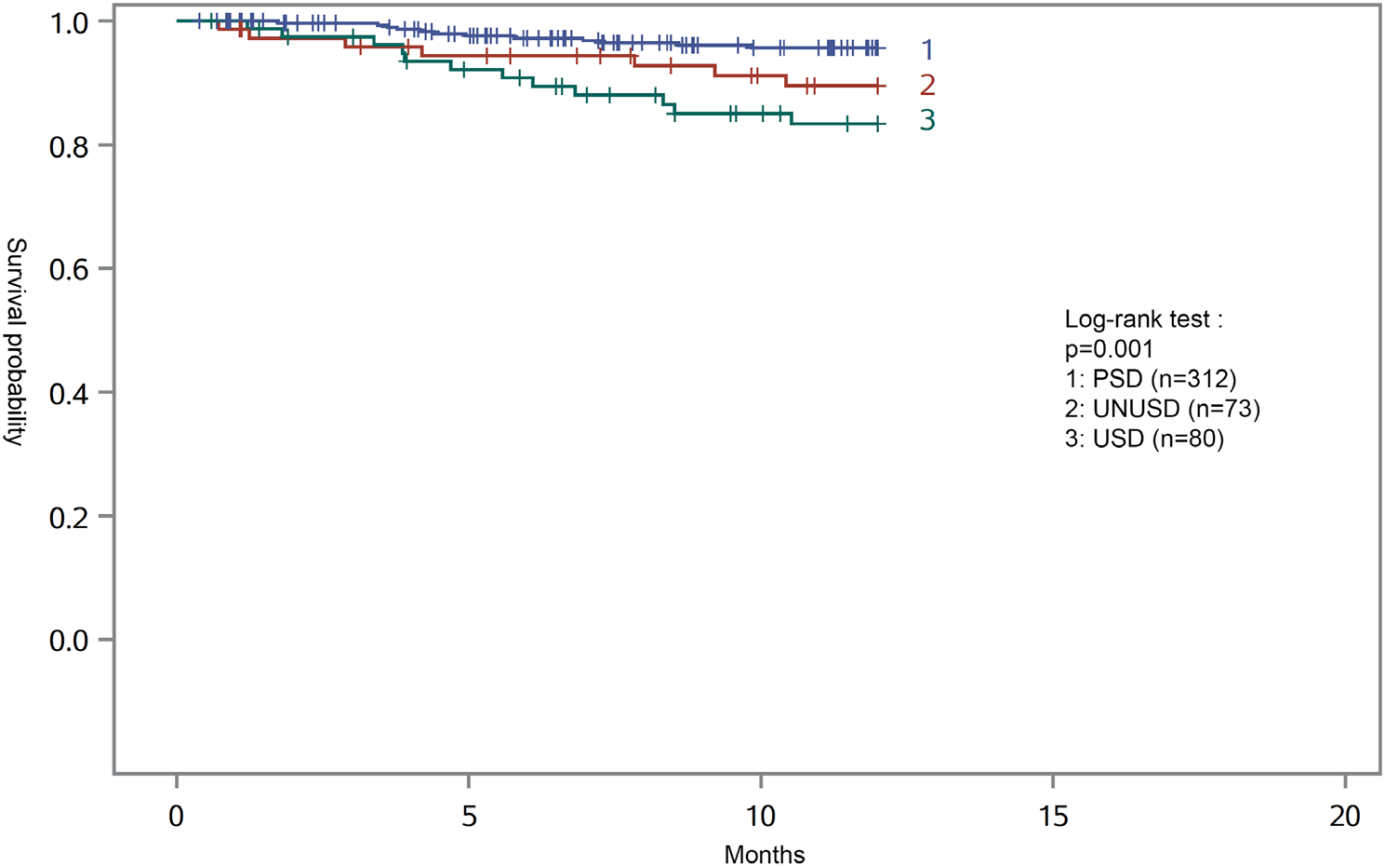
One-year survival after dialysis initiation in the different groups estimated with the Kaplan-Meier method (n = 465) PSD, planned start dialysis; UNUSD, unplanned non urgent start dialysis ; USD, urgent start dialysis

Concerning mortality during the first year of dialysis, we observed 31 deaths and half of deaths occurred before a median period of 4.7 months (IQR 3.6–7.8). We did not observe any significant difference between the two groups defined by REIN classification (7 (7.5%) vs. 24 deaths (6.5%) in USD and PSD respectively, p = 0.74). In contrast, according to our classification, survival analysis showed a correlation between dialysis starting context and mortality, USD patients being at higher risk of mortality with 12 deaths in this group (15%) vs. 7(9.6%) and 12(3.9%) in UNUS and PS respectively (p < 0.001). Survival curves estimated with Kaplan Meier method are shown in Fig. 1. Survival probabilities were 95.7%, 89.5% and 83.4% at one year respectively for PS, UNUS and US (p = 0.001).

The realization of KT in the first year after dialysis was more frequently observed in patients with planned dialysis start (0 vs. 1 (1.4%) vs. 22 (7.1%) in US, UNUS and PS respectively; p = 0.005). In contrast US were more at risk of dialysis withdrawal (7.5% for US vs. 4.1% and 1.9% for suboptimal and PS respectively, p = 0.03).

Univariable Cox regression (Table 3) showed only two factors associated with one-year mortality: start with a CVC (Hazard Ratio (HR) = 2.8; 95%CI [1.4–5.8]) and USD (HR = 3.2; 95%CI [1.5–6.5]). Because of a few number of deaths, no multivariable analysis was performed.

**Table 2:** Factors associated with one-year mortality, univariable analysis using a Cox proportional-hazard model (n = 465)

	USD (REIN classification)			
	Yes	No	*	Total
Planned	15 (16.0)	296 (80.4)	1	312
Unplanned non urgent	22 (23.4)	51 (13.8)		73
Urgent	57 (60.6)	21 (5.7)	2	80
Total	94	368	3	465

* Missing data in the REIN registry, USD: urgent start dialysis

**Table 3:** Factors associated with one-year mortality, univariable analysis using a Cox proportional-hazard model (n = 465)

	HR	CI	p value
Sex	1.29	0.63–2.65	0.49
Age (years)	1.02	0.99–1.05	0.17
BMI	1	0.95–1.06	0.96
Diabetes	1.67	0.82–3.38	0.16
Ischemic cardiomyopathy	1.74	0.69–4.65	0.23
cardiac failure	2.0	0.98–4.05	0.55
Current smoker	0.22	0.03–1.60	0.13
Stroke	1.69	0.65–4.38	0.17
COPD	0.42	0.1–1.76	0.24
Peripheral arteriopathy	0.9	0.39–2.10	0.82
IS	1.67	0.72–3.87	0.23
Cancer	1.03	0.44–2.38	0.95
CVC	3.24	1.59–6.61	0.001
RASB	0.6	0.29–1.25	0.17
Number of antihypertensive drugs	0.83	0.63–1.09	0.18
Fluid overload	1.36	0.6–3.06	0.46
Number of consultations prior dialysis starting	0.95	0.82–1.11	0.53
USD according our classification	3.17	1.54–6.52	0.002
USD according REIN classification	1.14	0.49–2.64	0.76

BMI: body mass index, CI: confidence interval COPD: chronic obstructive pulmonary disease, CVC: central venous catheter, HR: hazard ratio, IS: immunosuppression, PSD: planned start dialysis, RASB: renin-angiotensin-system blocker, UNUSD: unplanned non urgent start dialysis, USD: urgent start dialysis,

## Discussion

In our study, the USD rate were 17.2% and 20.3% according to our and REIN classification respectively. The national REIN registry recorded a 30% USD rate in 2018. The lower rate in our cohort is probably due to the fact that US without nephrology follow-up were not included. In our cohort, all patients were regularly followed by nephrologists. We included patients with a minimum of 2 consultations; the mean number of consultations was 5.44 ± 2.35 during the year prior dialysis. Indeed, late referral has already been described as a major risk factor for USD in several studies [7, 8]. This late referral is also associated with excess mortality [3]; as an example in the meta-analysis of Chan et al. in 2007, including 12 749 patients, the relative risk was 1.99 (95%CI [1.66–2.39] p < 0.0001) [8].

Concerning factors associated with USD, age was not a risk factor whatever the classification, this observation is in harmony with previous studies [9, 10]. Michel et al. in 2018, in a study based on REIN registry data, found similar results in a previous period (from 2006 to 2012) [10].

In our cohort, the two independent factors associated with USD were heart failure (OR = 1.95 95%CI [1.15–3.32], p < 0.01) and stroke (OR = 2.77 IC95% [1.41–5.47], p = 0.02). Heart failure is a common risk factor found in the literature [9–12]. This phenomenon could be explained by the fact that cardiac failure is associated with frailty and a high-risk of acute decompensation. Stroke is not a source of decompensation like chronic diseases such as diabetes or heart disease, but is rather a marker of frailty and precarious vascular condition.

We highlighted that the number of consultations was independently associated with a risk reduction of USD. This observation argues for the importance of early referral [13, 14].

The use of RASB in advanced chronic kidney disease is subject to debate. Although several studies showed a positive impact of this drug on the progression of CKD [15–18], this kind of treatment may be responsible of hyperkalemia or acute kidney injury in patients with CKD, potentially precipitating dialysis. RASB are associated in our study with the PSD. Interpretation of our observation is tricky. As the RASB data was collected at the dialysis start, hence, we may not exclude that some patients may have been taking a RASB formerly which was stopped before dialysis start by the nephrologist. Moreover, the RASB discontinuation may reflect a propensity of the patient to hyperkalemia or rapid kidney function decline. Anyway, we cannot conclude that RASB therapy is a protective factor for USD. A randomized clinical trial may answer this question, a work is currently in progress directed by Bhandary et al. [19].

Our study showed a different classification of USD with a few patients considered as US in REIN definition, and not in ours and conversely. The observed mismatch might be related to a very large heterogeneity of patients considered as US in REIN in terms of preparation to RRT. Whereas in our classification, the functionality and existence of the DA, represents the discriminant element. Some of US according to REIN could have been well prepared to dialysis start and might not have the same prognosis than those who were not prepared since they probably start RRT with better conditions.

In our cohort, we observed 31 deaths (6,7% rate of mortality) at one year after the beginning of dialysis. This rate is lower than national REIN registry data, which was around 15,9% in 2018. We can explain this difference because we did not include some type of patients like those who started RRT for heart failure refractory to drug therapy and the patients with late referral, who have worse outcomes. Comparisons of mortality in this cohort between PS and US according to REIN classification do not reveal any significant difference contrary to our definitions. We highlighted a significant increased mortality in US. Our classification led to more relevant groups in terms of survival. Although identified groups in the two classifications had different outcomes; US had the same profile in terms of comorbidity. This could reflect the influence of DA on outcomes. Indeed, a prospective multicenter Japanese cohort study on a population of 1 341 patients showed that the first DA when it was not a NAVF had an impact on mortality (HR = 1.60; p = 0.048), as well switching from a CVC to an NAVF (HR = 2.26; p = 0.003) and switching from CVC to PAVF (HR = 2.45; p = 0.001) [2]. The presence of a functional DA at dialysis initiation might constitute a marker of the quality of the preparation provided during follow-up prior to RRT and of the management of CKD-specific conditions.

In our study, we observed that more than a quarter of patients start RRT with an eGFR superior to 10 mL/min/1.73 m² while more than a quarter start with an eGFR inferior to 7 mL/min/1.73 m². The best timing to start dialysis has long been a subject of debate. The IDEAL study tried to answer this question but did not bring a definitive conclusion due to protocol deviations [20]. In fact, the lack of consensus concerning the optimal timing for dialysis start probably contributes to practice heterogeneity in this pre RRT prelude [21]. Thus, it may explain a part of USD. This situation is associated with an increased morbi-mortality and a higher cost[6]. The challenge for the nephrologist is to be able to organize the start as well as possible. Many other studies have tried to identify risk factors that could be associated with USD [5, 9, 10, 12, 22]. The difficulty to identify the determinants of USD is partly due to the absence of a consensual definition of emergency and to the heterogeneity of the populations studied [11].

However, our study had some limitations. It is a retrospective study with numerous missing data that sometimes limit the weight of the results. Because of a low mortality, we met a lack of power; that did not permit us to perform multivariable survival analyses to identify risk factor of mortality. Since our cohort focused on patients followed by nephrologists, our results cannot be generalized to another population. A cohort study with a prospective follow up and a larger population would allow us to confirm our results.

Our definition of urgent-start, based on the existence of a functional DA, and the REIN definition showed US with similar comorbid conditions. The two main risk factors independently associated with emergency dialysis were cardiac failure and stroke. US according our classification had a significant lower one-year survival on dialysis.

## Electronic supplementary material

Below is the link to the electronic supplementary material.

Supplementary Material 1

## Data Availability

The datasets generated and analyzed during the current study are available from the corresponding author on reasonable request.

## Code Availability

NA.

## Declarations

All methods were performed in accordance with the relevant guidelines and regulations (Declaration of Helsinki).
