# Supplementary material for: Determinants of urgent start dialysis in a chronic kidney disease cohort followed by nephrologists
Source: BMC Nephrol. 2023 Jun 27;24:190. doi: 10.1186/s12882-023-03222-1 (PMC10304309; doi:10.1186/s12882-023-03222-1)
Supplement: Supplementary file 1 — Supplementary Material 1 [file 12882_2023_3222_MOESM1_ESM.docx]

| **Supplementary Table 1: Biological parameters at dialysis initiation according to our classification** | | | | |
| --- | --- | --- | --- | --- |
| USD according our classification | | | |  |
| Unplanned urgent | Unplanned non urgent | Planned | p value |  |
| Hemoglobin (g/dL, 4*) | 9.6 ±1.6 | 9.6 ±1.6 | 10.5 ±1.5 | <0.0001 |
| Creatinine (µmol/L) | 677.1 ±276.1 | 603.2 ±175.8 | 573.8 ±185.6 | 0.004 |
| Urea (mmol/L) | 38.4 ±11.5 | 36.1 ±11.0 | 30.6 ±9.7 | <0.0001 |
| Sodium (mmol/L, 2*) | 138.0 ±4.1 | 137.0 ±5.0 | 139.0 ±8.0 | 0.0006 |
| Potassium (mmol/L, 1*) | 4.6 ±0.9 | 4.2 ±0.6 | 4.4 ±0.6 | 0.002 |
| Bicarbonates (mmol/L, 2*) | 18.6 ±5.9 | 20.2 ±4.5 | 21.2 ±4.2 | 0.0004 |
| Albumin (g/L, 15*) | 27.9 ±6.2 | 31.4 ±5.4 | 33.6 ±6.0 | <0.0001 |
| Calcium (mmol/L, 5*) | 2.5 ±2.1 | 2.2 **±**0.3 | 2.3 ±1.1 | 0.35 |
| Phosphorus (mmol/L, 13*) | 2.08 ±0.68 | 1.83 ±0.49 | 1.72 ±0.51 | <0.0001 |
| CRP (mg/L, 46*) | 20.7 ±44.9 | 21.0 ±49.5 | 6.9 ±17.1 | 0.07 |
| Fluid overload (83*) | 50 (72.5) | 30 (54.6) | 113 (43.8) | 0.0001 |
| *Missing data |  |  |  |  |

USD: Urgent start dialysis, CRP: C-reactive protein

**Supplementary Table 2: Factors associated with USD* according to our classification (logistic regression, c-statistic=0.73)**

|  | OR |  | CI | p value |  |
| --- | --- | --- | --- | --- | --- |
| Stroke | 2.76 |  | 1.41 – 5.43 | 0.02 |  |
| Cardiac failure | 1.78 |  | 1.07 – 2.96 | 0.003 |  |
| Number of consultations | 0.73 |  | 0.64 - 0.83 | 0.02 |  |
| *USD ( urgent start dialysis) *vs.* the others, OR: odds ratio, CI: confidence interval | | | | | |
